# Research on Quality Markers of Guizhi Fuling Prescription for Endometriosis Treatment Based on Gray Correlation Analysis Strategy

**DOI:** 10.3389/fphar.2020.588549

**Published:** 2021-01-12

**Authors:** Jinpeng Chen, Xiaohong Gai, Xu Xu, Yi Liu, Tao Ren, Suxiang Liu, Ting Ma, Chengwang Tian, Changxiao Liu

**Affiliations:** ^1^ State Key Laboratory of Drug Delivery and Pharmacokinetics, Tianjin, China; ^2^ Tianjin Key Laboratory of TCM Quality Markers, Tianjin, China; ^3^ Tianjin Institute of Pharmaceutical Research, Tianjin, China; ^4^ University of Traditional Chinese Medicine, Office of Academic Research, Jinan, China

**Keywords:** gray correlation analysis, endometriosis, traditional Chinese herb formulations, Guizhi Fuling prescription, Q-markers

## Abstract

Guizhi Fuling prescription (GFP), a prestigious prescription of traditional Chinese medicine (TCM) recorded in “Jingui Yaolue,” was composed of five Chinese medicines, including Moutan Cortex, Paeoniae Radix Alba, Persicae Semen, Poria Cocos, and Cinnamomi Ramulus. It was used for the treatment of endometriosis, primary dysmenorrhea, and blood stasis for centuries. However, its Quality Markers of treating endometriosis have not been clearly elucidated. In this study, a rapid ultraperformance liquid chromatography/quadrupole time-of-flight tandem mass spectrometry (UPLC/Q-TOF-MS/MS) method was established for Quality Markers investigation on GFP, and a total of 50 potentially bioactive constituents including triterpenoids, paeoniflorin and its derivatives, phenolic acids, and other species were identified based on their retention time, fragmentation pattern, and accurately measured mass value. Furthermore, regularity of recipe composition and gray correlation analysis revealed that all of the characteristic peaks contributed to the treatment of endometriosis. The relative correlation degrees were greater than 0.6. Among them, peaks 1 and 10, which were most closely correlated to the endometriosis, were identified as amygdalin and cinnamic acid. Finally, all of the active ingredients were molecularly docked with proteins associated with endometriosis by Schrodinger method. Among them, amygdalin, cinnamic acid, paeonol, gallic acid, and paeoniflorin had the lower binding energies. It was proposed that these constituents could be directed at Quality Markers for GFP. Thus, the integrated approach describing for revealing Quality Markers of GFP could be expected to provide a method for quality evaluation.

## Introduction

As one of the ancient medicine systems, traditional Chinese medicine (TCM) has been used to treat various diseases for thousands of years. The plentiful experience was accumulated, and unique theories were formed in the long historical clinical practice (Zhao et al., 2009). Traditional Chinese herb formulations (TCHF) are the essence of the TCM, and hundreds of different natural components, both volatile and nonvolatile, are present in TCHF and play different important roles in their therapeutic effects. On account of its highly complex chemical constituents, TCM has always been bottleneck hindering its development in quality control and therapeutical mechanism research (Yang et al., 2017). Recent years, the quality standards of TCM were established according to their major or special chemical constituents in Chinese Pharmacopeia. Some of the TCM even have no clear index components instead of the character test, such as Curcumae Radix (the dried root of *Curcuma longa* L.) and Dioscoreae Rhizoma (the dried rhizoma of *Cyperus rotundus* L.). Thus, the content of the specified chemical constituents can only be ensured by this method. Whether these specific chemical components are related to or directly related to the different effects of TCM is remain suspicious.

A lot of work has been done, in order to promote the development of quality management of traditional Chinese medicine (Xiao et al., 2012; Guo et al., 2015; Long et al., 2015). However, using ranges and limitation of each method and the individual differences of each TCM make the strategy difficult to follow. Therefore, a commanding, universal, and standardized TCM quality control research strategy was urgently needed to be developed.

Fortunately, in order to develop the research of TCM quality and improve the consistency of TCM quality, the concept of Quality Marker (Q-Marker) was proposed by Liu et al. (2016). The Q-Marker is a practical value concept for controlling the quality, efficacy, and safety of original herbs, extracts, and preparations for TCM. The candidates for Q-Markers should be equipped with the following conditions: 1) The candidates for Q-Markers should exist in the whole process or be traceable and transferable in the process of TCM and related to the efficacy. 2) The candidates for Q-Markers should be qualitatively and quantitatively analyzed. 3) The candidates for Q-Markers should have biological effect and be traced to the original herbs. 4) The candidates for Q-Markers should be suitable for the theory and practice of TCM (Liu et al., 2016; Liu et al., 2017). In recent years, lots of studies on Q-Marker have been published (Liu et al., 2017; Wang et al., 2017; Tang et al., 2018; Wu et al., 2018; Zhang et al., 2018; Gao et al., 2019; Li and Ding, 2019; Wang et al., 2019; Yang et al., 2019; Zhai et al., 2019). However, it is still a huge challenge how to discover and validate the Q-Markers.

**FIGURE 1:**
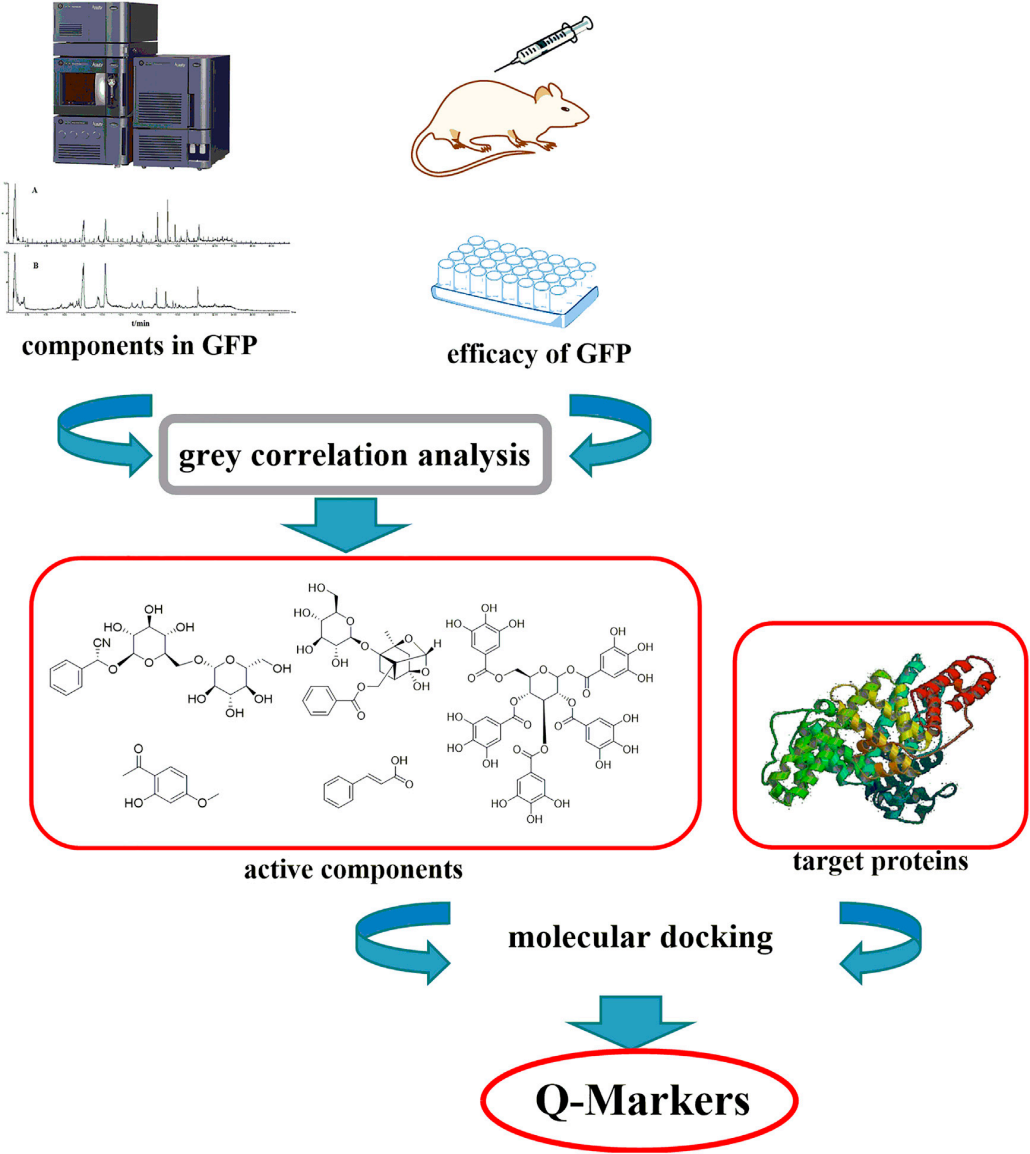
The strategy of Q-Markers discovery for GFP by gray correlation analysis method.

Guizhi Fuling prescription (GFP), an important and widely used Chinese prestigious prescription recorded in “Jingui Yaolue,” was used for the treatment of endometriosis, primary dysmenorrhea, and blood stasis for centuries. The prescription is composed of *Poria Cocos* (the dried sclerotum of the fungus (*Schw*.)) *Wolf* (*Poria*), *Moutan Cortex* (the dried root bark of *Paeonia suffruticosa* Andrews), *Cinnamomi Ramulus* (the dried twig of *Cinnamomum cassia* (L.) J. Presl), *Persicae Semen* (the dried mature seed of *Prunus persica* (L.) Batsch), and *Paeoniae Radix* Alba (the dried root of *Paeonia lactiflora* Pall.). Although the ingredients of each comprising herb of GFP have been studied in previous reports (Tai et al., 1993; Lin et al., 1996; Kamiya et al., 1997; Fukuda et al., 2003; Choi et al., 2011), its Q-Markers of treating endometriosis has not been clearly elucidated. Therefore, it is necessary to develop a strategy to identify and validate the Q-Markers of treating endometriosis in GFP. In this work, a rapid method was established for Q-Markers investigation on GFP (Figure 1). The results proposed that amygdalin, cinnamic acid, paeonol, gallic acid, and paeoniflorin could be directed at Q-Markers for GFP. Thus, the integrated approach describing for revealing Q-Markers of GFP could be expected to provide a method for quality evaluation.

## Materials and Methods

### Plant Material

The *Poria Cocos* (the dried sclerotum of the fungus (*Schw*.)) *Wolf* (*Poria*), *Moutan Cortex* (the dried root bark of *Paeonia suffruticosa* Andrews), *Cinnamomi Ramulus* (the dried twig of *Cinnamomum cassia* (L.) J. Presl), *Persicae Semen* (the dried mature seed of *Prunus persica* (L.) Batsch), and *Paeoniae Radix* Alba (the dried root of *Paeonia lactiflora* Pall.) were purchased from Tianjin traditional Chinese Medicine Decoction Pieces Factory Co., Ltd., and identified by Researcher Liu Suxiang. The voucher specimens (PCGFP-2018, MCGFP-2018, CRGFP-2018, PSGFP-2018, and PRGFP-2018) were stored at Tianjin Institute of Pharmaceutical Research, China.

### Animals, Chemicals, and Reagents

The animal experiments were conducted according to the Guideline of Animal Experimental, and a total of 36 adult female SD rats (body weight: 240–250 g) were provided by Beijing Huafukang Biotechnology Co., Ltd. Moreover, the animal experiment protocol has been approved by the Animal Ethics Committee of Huafukang Biotechnology Co., Ltd.

Progesterone capsules were purchased from Zhejiang Xianju Pharmaceutical Co., Ltd. Iodine was purchased from Beijing Changjiangmai Pharmaceutical Technology Co., Ltd. Estradiol valerate tablets were purchased from DELPHARM Lille S.A.S. All organic solvents used in this study were HPLC-grade and purchased from Concord Technology Co., Ltd. Pure distilled water was purchased from Watsons.

### Sample Preparation

The whole prescription sample was prepared according to the Chinese Pharmacopeia 2015 Volume I. Moutan Cortex (240 g) was distilled to afford volatile constituents. The residues were mixed with Cinnamomi Ramulus (240 g), Paeoniae Radix Alba (240 g), Persicae Semen (240 g), and Poria Cocos (48 g), and then they were refluxed with 90% EtOH and pure water twice (1:10, w/v, 2 h each). After evaporation of solvent in vacuo, the crude extract was mixed with the powder of Poria Cocos (192 g) and the volatile constituents extracted from the Moutan Cortex. Samples for regularity of recipe composition (GFP-B1 ∼ B8) were prepared as the above method, except for the assigned herbs. The ingredients of each sample were shown in Table 1.

**TABLE 1:** Ingredients and dosage of administration of each group.

No.	Ingredients	Dosage of administration (mg/kg)
NCG	—	—
MCG	—	—
PG	Progesterone	20.0
GFP-A1G	CR, PC, MC, PS, PR	77.0
GFP-B1G	CR, MC, PS, PR	32.0
GFP-B2G	CR, PC, PS, PR	58.0
GFP-B3G	CR, PC, MC, PS	69.0
GFP-B4G	CR, PS, PR	19.0
GFP-B5G	CR, MC, PS	31.0
GFP-B6G	CR, PC, PS	51.0
GFP-B7G	PC, PS	10.2
GFP-B8G	PC, MC, PS, PR	72.0

CR: Cinnamomi Ramulus; PC: Poria Cocos; MC: Moutan Cortex; PS: Persicae Semen; PR: Paeoniae Radix Alba.

### Components Identification in GFP

In this study, an UPLC/Q-TOF-MS/MS method was developed to clarify the components existing in GFP. Approximately 0.2 g of the whole prescription sample (GFP-A1) was refluxed with 25 ml of 70% methanol for 30 min. Then the 70% methanol extraction solution was centrifuged for 15 min at 3,000 rpm. Next, the supernatant was filtered through a 0.22 μm membrane before being injected to the UPLC system, and then an aliquot of 3 μl filtrate was injected to the UPLC system for analysis. For UPLC/Q-TOF-MS/MS analysis, the compounds were separated through a Waters Acquity UPLC BEH C18 column (1.7 μm, 2.1 × 100 mm) using 0.1% aqueous formic acid (A) and acetonitrile (B) as the mobile phase. The method of elution was gradient elution, and the optimized program was as follows: 99% A at 1–3 min, 99–92% A at 3–6 min, 92–88% A at 6–10 min, 88–84% A at 10–13 min, 84–75% A at 13–18 min, 75–60% A at 18–23 min, 60–55% B at 23–25 min, and eventually equilibration for 5 min. The flow rate was fixed at 0.4 ml/min, and the column temperature was maintained at 35°C.

A Xevo G2-S Q TOF MS (Waters MS Technologies, Manchester, United Kingdom) equipped with an ESI interface was performed to achieve the mass spectrometry analysis. The mass spectrum parameters were set as follows: positive and negative ion modes were used for detection, and the capillary voltages were 3.0 and 2.0 kV, respectively. In addition, the desolvation temperature and the source temperature were set to 350 and 110°C, respectively. The desolvation gas flow was 800 L/h. In order to analyze the components accurately and completely, the full scan of mass data was achieved in the mass range of *m*/*z* from 50 to 1,500 Da. Moreover, the mass was corrected by leucine encephalin via a lock spray interface and monitoring a reference ion at 554.2615 Da for negative ion mode and at 556.2771 Da for positive ion mode. The system was controlled by Masslynx 4.1.

### UPLC-PDA Analysis for Regularity of Recipe Composition Samples

The analysis of regularity of recipe composition samples (GFP-B1 ∼ B8) was performed by the Waters Acquity UPLC (Waters H-class, United States) with PDA detector. The gradient elution program for mobile phase, flow rate, chromatography column, and column temperature were set as UPLC/Q-TOF-MS/MS method. The optimal absorbed wavelength was selected as 220 nm according to the multiple chromatographic peaks and favorable resolution.

### Animal Experiments

All rats were bred in animal center, and the temperature was maintained at 25 ± 2°C with a relative humidity of 50 ± 10%. Moreover, all the rats were reared in natural light cycle for 7 days, irrigated with water every other day, and fasted for 12 h before the experiment.

Thirty-six experimental rats were divided into 12 groups, each group including three rats. Detailed groups were shown as follows: positive group (PG), model control group (MCG), normal control group (NCG), the whole prescription group (GFP-A1G), and regularity of recipe composition groups (GFP-B1G ∼ GFP-B8G). According to the previous reports, the endometriosis model was performed by autotransplantation. Specifically, rat was subjected to 10% chloral hydrate by subcutaneous injection and fixed on the operating table. Then, its abdomen was disinfected and scissored to find the uterus. The middle uterus was separated through ligating the point 1 cm from the right of ovary and 1 cm from the uterine bifurcate. After cutting the uterus, the endometrium was removed and sewn near the left ovary. At the end of the experimental period, its abdomen was sutured. Estradiol was used to induce the growth of ectopic tissue for 1 week after surgery. All rats except for NCG were subjected to the surgery above. Each group was administered with the corresponding drug once a day for 1 month. The content of crud drug for each group was equal. The dosage of each group was shown in Table 1.

The blood for each group was collected in tubes and separated by centrifugation for 15 min at 2,500 rpm. The supernatant was collected and then stored at −80°C and moved to room temperature before bioanalysis. Ectopic tissues were separated to record their volume.

### Biochemical Analysis

After 1 month of treatment, the level of CA-125 in the serum was detected through radioimmunoassay. The level of CA-125 can be used to evaluate the curative effect of endometriosis due to its sensibility and specificity.

### Selection of GFP Ingredients, Endometriosis Targets, and Docking Experiment

The principle of selecting ingredients for docking is based on the following aspects. Firstly, the selecting ingredients must be the main components in GFP. Then, it must be absorbed into the blood. According to the principle above and previous reports, 13 compounds, including paeonol, cinnamic acid, cinnamaldehyde, paeoniflorin, gallic acid, amygdalin, benzoic acid, pentagalloyl glucose, albiflorin, benzoylpaeoniflorin, pachymic acid, dehydropachymic acid, and dehydrotumulosic acid, were considered for docking study. After that, 41 proteins related to endometriosis were discovered from RCSB (http://www.rcsb.org/pdb/home/home.do) and TTD (http://bidd.nus.edu.sg/group/cjttd/). The corresponding PDB IDs of these proteins were discovered on Uniport (http://www.uniport.org/). Subsequently, ligands were constructed using Schrodinger Suites (Maestro Version 11.8.012, MMshare Version 4.4.012, Schrodinger, LLC, New York, 2018) with CHARMm force field parameters. Flexible docking and grid-based scoring were implemented in the CDOCKER protocol. Finally, the highest score of conformation for each receptor was ticked off by the “-CDOCKER ENERGY” for followed analysis. The docking affinity depends on the absolute value of the docking score between the ligand and receptor. The larger the absolute value, the higher the docking affinity. Usually, the absolute value of the docking fraction of the ligands is ≥5.0, and the compounds were considered to be an active compound.

## Results and Discussion

### Components Identification in GFP

#### Construction of Ingredients Database for GFP

The chemical constituents of five herbs and GFP were collected by reviewing and analyzing relevant literatures to build a database for GFP. The database focuses on phytochemical ingredients, including English names, molecular formulas, structures, characteristic fragment ions, CloP values, and CAS number.

#### MassLynx Processing Approach for GFP Components

Analysis mass data processing was implemented by MassLynx 4.1. The narrow mass window of EICs was 0.01 Da, the fraction isotope abundance value was set as 1.0, and the calculation of ECs with mass errors was controlled within 10 ppm. The EICs can be used to distinguish and identify the mixed peaks which had the similar retention time. The target ingredients can be identified based on accurately measured mass value, ECs, characteristic fragment ions, and retention time by comparing with the database for GFP.

#### Analysis of Ingredients in GFP

**FIGURE 2:**
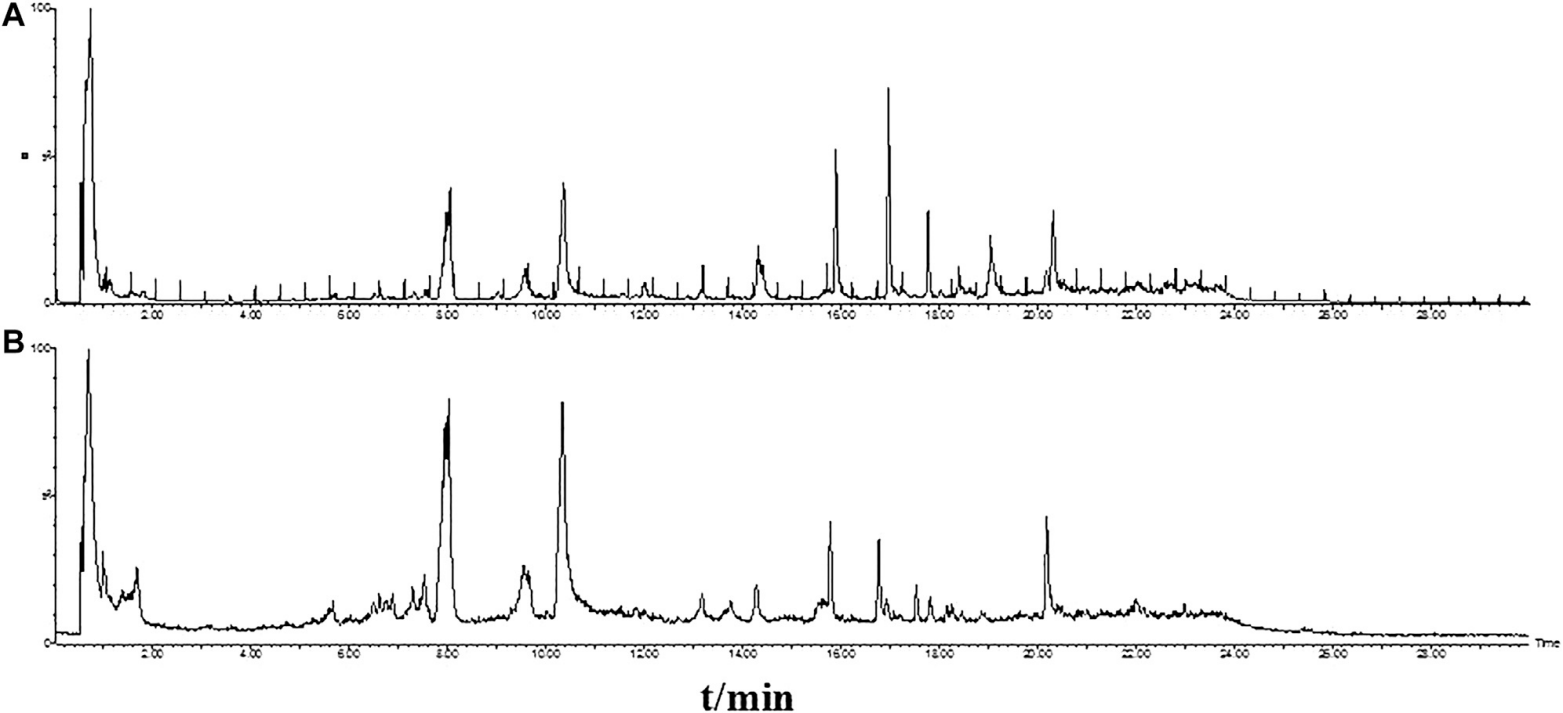
The base peak intensity (BPI) chromatograms of GFP extract by UPLC/Q-TOF-MS/MS at positive **(A)** and negative **(B)** ion modes.

**FIGURE 3:**
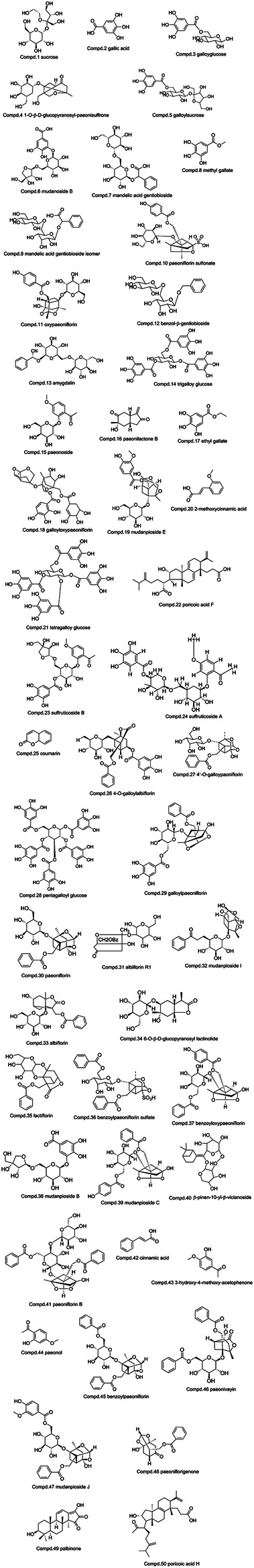
Chemical structures identified in GFP.

The base peak intensity (BPI) chromatograms of GFP extract at positive and negative ion modes were shown in Figure 2. In this study, a total of 50 components were identified or tentatively characterized, including 20 phenolic acids, 21 paeoniflorins and its derivatives, 2 triterpenoids, and 7 other species, such as sucrose and coumarin. Three of them were from Cinnamomi Ramulus, 2 of them were from Poria Cocos, 25 of them were from Moutan Cortex, 26 of them were from Paeoniae Radix Alba, and 4 of them were from Persicae Semen. Eleven of them were shared with Moutan Cortex and Paeoniae Radix Alba. Most of them can be responded in the negative ion mode, instead of positive mode. Therefore, the identification of compounds was mainly based on the analysis of negative ion mode and supplemented by the positive mode and compared with the database for GFP and literatures, including the fragmentation behavior and retention time. The database made the task convenient and significant even in the absence of reference standards. However, the presence of isomers in natural products and subtle differences in mass spectra made some compounds difficult to distinguish. Therefore, the polarity and electronic effect should be considered. It was especially critical for screening the various components fast and firmly to calculate correct monoisotopic mass values of quasi-molecular ions and rational fragment ions. Many isomers inevitably exist in complex natural products, and they were difficult to distinguish because of the tiny difference of the mass spectra. Therefore, in order to distinguish and identify compounds, the electronic effects and their hydrophilicity should also be considered. The detailed information including chemical formula, mass values of quasi-molecular ions, botanical source, and mass error for the identified compounds was shown in Table 2. In addition, their chemical structure information was shown in Figure 3.

**TABLE 2:** Identification results of chemical composition in Guizhi Fuling prescription.

No.	t_R_ (min)	Identification	Molecular formula	[M-H]^−^			[M + H]^+^			Other adduct ions	Sources
				Meas.	ppm	MS/MS	Meas.	ppm	MS/MS		
1	0.68	Sucrose	C_12_H_22_O_11_	341.1118	9.9	327.0957, 191.0580, 165.0423, 96.9611				387.1172 [M + HCOO]^−^, 683.2292 [2M-H]^−^, 1,025.3497 [3M-H]^−^	All
2	1.67	Gallic acid	C_7_H_6_O_5_	169.0156	11.2	125.0255, 123.0101					PR, MC
3	1.90	Galloyglucose	C_13_H_16_O_10_	331.0681	4.8	271.0412, 235.9294, 169.0158, 125.0264				663.1614 [2M-H]^−^	PR, MC
4	3.17	1-O-β-D-glucopyranosyl-paeonisuffrone	C_16_H_24_O_9_	359.1370	7.7	179.0749, 145.9326, 116.9294, 101.9421				405.1439 [M + HCOO]^−^	PR
5	3.64	Galloylsucrose	C_19_H_26_O_15_	493.1208	3.0	352.8681, 315.0728, 146.9393, 116.9297					PR
6	4.12	Mudanoside B	C_18_H_24_O_14_	463.1103	3.2	403.0903, 315.0799, 146.9396, 116.9295					MC
7	6.00	Mandelic acid gentiobioside	C_20_H_28_O_13_	475.1479	5.6	445.1021, 431.1604				951.3041 [2M-H]^−^	PS
8	6.49	Methyl gallate	C_8_H_8_O_5_	183.0310	9.2	116.9304					PR, MC
9	6.61	Mandelic acid gentiobioside isomer	C_20_H_28_O_13_	475.1503	10.7	349.0634, 289.0735, 175.0634				951.3049 [2M-H]^−^	PS
10	6.87	Paeoniflorin sulfonate	C_23_H_28_O_13_S	543.1208	6.6	349.0626, 289.0733					PR
11	7.28	Oxypaeoniflorin	C_23_H_28_O_12_	495.1539	7.2	443.1961,293.1276				991.3098 [2M-H]^−^	MC
12	7.51	Benzol-β-gentiobioside	C_19_H_28_O_11_	431.1585	7.4	379.1601, 235.9279, 146.9398				477.1653 [M + HCOO]^−^	PS
13	8.01	Amygdalin	C_20_H_27_NO_11_	456.1537	6.7	323.1008, 221.0694	458.1658	0.8	378.0248, 325.1126, 145.0506	502.1590 [M + HCOO]^−^, 475.1922 [M + NH4]^+^	PS
14	9.05	Trigalloy glucose	C_27_H_24_O_18_	635.0916	5.0	445.1397, 235.9276, 145.9328, 116.9296					PR, MC
15	9.29	Paeonoside	C_15_H_20_O_8_	327.1112	9.4	235.9283, 165.0587, 116.9298					MC
16	9.57	Paeonilactone B	C_10_H_12_O_4_				197.0818	2.0	151.0756, 133.0666		PR
17	9.64	Ethyl gallate	C_9_H_10_O_5_	197.0474	12.1	169.0148					PR
18	10.01	Galloyloxypaeoniflorin	C_30_H_32_O_16_	647.1647	5.4	631.1637, 495.1558, 400.1546					MC
19	10.32	Mudanpioside E	C_24_H_30_O_13_	525.1654	8.7	479.1607, 449.1498					MC
20	10.35	2-Methoxycinnamic acid	C_10_H_10_O_3_				179.0714	3.3	151.0766, 135.0820	197.0825 [M + H + H_2_O]^+^	CR
21	11.83	Tetragalloy glucose	C_34_H_28_O_22_	787.1025	3.9	605.2534,555.1767, 479.1571, 449.1501, 285.0781, 137.0259					PR, MC
22	12.00	Poricoic acid F	C_31_H_46_O_5_	497.3373	2.1	451.3305, 352.8586, 145.9314					PC
23	12.40	Suffruticoside B	C_27_H_32_O_16_	611.1665	8.6	525.1668, 449.1493, 262.8580					MC
24	12.70	Suffruticoside A	C_27_H_32_O_16_	611.1668	9.1	525.1647, 449.1497, 352.8613					MC
25	12.89	Coumarin	C_9_H_6_O_2_				147.0444	−1.3			CR
26	13.08	4-O-galloylalbiflorin	C_30_H_32_O_15_	631.1724	9.6	525.1632, 440.0487, 190.9306					PR, MC
27	13.17	4′-O-galloypaoniflorin	C_30_H_32_O_15_	631.1715	8.2	611.1674, 497.3344					PR, MC
28	13.76	Pentagalloyl glucose	C_41_H_32_O_26_	939.1105	0.1	769.0944, 631.1730, 495.5153, 469.0565				469.0569 [M-2H]^−^	PR, MC
29	14.85	Galloylpaeoniflorin	C_30_H_32_O_15_	631.1714	9.1	525.1666, 479.1596, 352.8510					PR, MC
30	15.60	Paeoniflorin	C_23_H_28_O_11_	479.1600	9.8	449.1487, 435.1320, 327.1113, 165.0583, 121.0305				525.1644 [M + HCOO]^−^	PR
31	15.63	Isopaeniflorin	C_23_H_28_O_11_	479.1586	6.8	365.2029, 283.0804				525.1649 [M + HCOO]^−^, 959.3173 [2M-H]^−^	PR
32	15.66	Mudanpioside I	C_23_H_28_O_11_	479.1589	7.5	352.8579,283.0883				525.1663 [M + HCOO]^−^,959.3514 [2M-H]^−^	MC
33	15.67	Albiflorin	C_23_H_28_O_11_	479.1553	7.5	408.2122, 283.0836				525.1663 [M + HCOO]^−^, 959.3514 [2M-H]^−^	PR
34	15.70	6-O-β-D-glucopyranosyl lactinolide	C_16_H_26_O_9_	361.1744	6.7	283.0877, 116.9303	363.1523	−3.6		723.5091 [2M-H]^−^, 381.0792 [M + H_2_O + H]^+^	CR
35	16.01	Lactiflorin	C_23_H_26_O_10_	461.1473	5.4	339.1113, 146.9389				507.1547 [M + HCOO]^−^	PR
36	16.28	Benzoylpaeoniflorin sulfate	C_30_H_32_O_14_S	647.1472	5.7	425.2217, 352.8545, 235.9292, 16.9387					PR, MC
37	16.92	Benzoyloxypaeoniflorin	C_30_H_32_O_13_	599.1827	10.3	453.0613, 352.8582, 190.9304				1,199.3538 [2M-H]^−^	MC
38	17.22	Mudanpioside B	C_31_H_34_O_14_	629.1913	6.8	583.1865, 553.1748, 535.1623					MC
39	17.80	Mudanpioside C	C_30_H_32_O_13_	599.1818	8.8	352.8611, 299.0944				1,119.3649 [2M-H]^−^	MC
40	18.26	β-Pinen-10-yl-β-vicianoside	C_21_H_34_O_10_	445.2109	7.8	352.8583, 293.0902, 145.9323				491.2164 [M + HCOO]^−^	PR
41	18.46	Paeoniflorin B	C_36_H_42_O_17_	745.2390	6.1	599.1849, 505.2337, 421.1350, 289.2187				791.2487 [M + HCOO]^−^	MC
42	19.04	Cinnamic acid	C_9_H_8_O_2_				149.0600	−2.0	121.0660	167.0715 [M + H + H_2_O]^+^	CR
43	19.06	3-Hydroxy-4-methoxy-acetophenone	C_9_H_10_O_3_				167.0716	4.7	149.0600, 125.0607		MC
44	19.11	Paeonol	C_9_H_10_O_3_				167.0715	4.1	149.0613, 125.0609		PR
45	20.18	Benzoylpaeoniflorin	C_30_H_32_O_12_	583.1865	8.4	553.1745, 535.1650	585.1964	−1.3		629.1917 [M + HCOO]^−^, 1,167.3807 [2M-H]^−^, 607.1790 [M + Na]^+^	PR, MC
46	20.32	Paeonivayin	C_30_H_32_O_12_	583.1847	5.3	553.1761, 535.1705, 449.2768				629.1916 [M + HCOO]^−^, [M + NH_4_]^+^, 607.1747 [M + Na]^+^	PR
47	20.40	Mudanpioside J	C_31_H_34_O_14_	629.1938	10.8	583.1837, 553.1785					MC
48	20.53	Paeoniflorigenone	C_17_H_18_O_6_				319.1169	-4.1	301.1056, 267.0834, 249.0742, 197.0799, 186.0545		PR
49	22.36	Palbinone	C_22_H_30_O_4_	357.2066	−6.4	329.2330, 168.9915				329.2330 [m-CO-H]^−^	PR
50	23.58	Poricoic acid H	C_31_H_48_O_5_	499.3463	8.0	329.2371, 313.2409, 237.1515, 168.9939					PC

#### Identification of Phenolic Acids in GFP

Phenolic acids, a relatively common species in nature, were found in many herbs. In this work, a total of 20 phenolic acids and their glycosides were characterized, including compounds **2**–**3**, **5**–**9**, **12**, **14**–**15**, **17**, **20**–**21**, **23**–**24**, **28**, **38**, **42**–**44**. Most of them could yield the quasi-molecular ions [M-H]^−^. Moreover, compounds with galacyl fragment usually produced [M-H-COO]^−^, [M-H-H_2_O-COO]^−^ ions. These phenolic acids mainly came from Moutan Cortex and Paeoniae Radix Alba, and few of them came from Cinnamomi Ramulus and Persicae Semen.

#### Identification of Paeoniflorin and Its Derivatives in GFP

Paeoniflorin and its derivatives were the major phytochemical constituents in Moutan Cortex and Paeoniae Radix Alba. The characteristic structure was a pinane skeleton, which looked like a cage. Moreover, it was reported that mono-glucose moiety and esterified aromatic acid group, such as p-hydroxybenzoic acid, benzoic acid, p-methoxybenzoic acid, vanillin acid, and gallic acid, were also attached to the pinane skeleton (Zhang and Zhang, 2008). A total of 21 paeoniflorins and their derivatives were identified in this study, including compounds **4**, **10**–**11**, **18**–**19**, **26**–**27**, **29**–**33**, **35**–**37**, **39**, **41**, **45**–**48**. The compounds showed sensitive response in positive ion and negative ion mode, and high abundances of quasi-molecular ions [M + HCOO]^−^, [M-H]^−^, [2M-H]^−^ were observed. For the fragment patterns, [M-CH_2_O]^−^, [M-benzoic acid]^−^ were usually produced. Paeoniflorin was used as an example to illustrate the process of compound identification. The quasi-molecular ion [M-H]^−^ at *m/z* 479 that corresponded to the formula C_23_H_28_O_11_ was compound **30**. The MS^2^ fragmental ions of compound **30** at *m/z* 449 were obtained due to the loss of CH_2_O (30 Da) group. *m/z* 121 was the typical benzoic acid fragment ion. The quasi-molecular ion could also lose a benzoic acid and CH_2_O group to produce *m/z* 327. In addition, it was observed that there was another characteristic ion *m/z* 165 since the loss of glucose group from the ion at *m/z* 327. Therefore, compound **30** was identified as paeoniflorin. These compounds were mainly derived from Moutan Cortex and Paeoniae Radix Alba.

#### Identification of Triterpenoids in GFP

Two triterpenoids were identified in this study, including poricoic acid F (**22**) and poricoic acid H (**50**). Both of them could yield the quasi-molecular ions [M-H]^−^. They were derived from Poria Cocos.

#### Identification of Other Ingredients in GFP

In addition, other 7 ingredients including sucrose (1), amygdalin (13), paeonilactone B (16), coumarin (25), 6-O-β-D-glucopyranosyl lactinolide (34), β-pinen-10-yl-β-vicianoside (40), and palbinone (49) were identified by comparing MS data and retention behavior with database and literatures. Most of them were derived from Paeoniae Radix Alba, Cinnamomi Ramulus, and Persicae Semen.

#### Effect of GFP on Endometriosis Rats

The volume of ectopic tissue and score for different groups were shown in Table 3. It was observed that the ectopic tissue in the model control group (MCG) grew larger when compared with the normal control group (NCG). However, ectopic tissues in the whole prescription group (GFP-A1G) and regularity of recipe composition groups GFP-B1G, GFP-B4G, GFP-B5G, and GFP-B7G were significantly reduced, which were most obvious in the GFP-A1G and GFP-B4G.

**TABLE 3:** Endometrial tissue volume after administration.

No.	Volume (mm³)	*p*	Score
	X		
NCG	18.9	—	—
MCG	160.8^##^	—	—
PG	44.6**	0.007	4
GFP-A1G	20.2**	0.001	2
GFP-B1G	58.8**	0.035	6
GFP-B2G	114.1	0.322	9
GFP-B3G	90.3	0.142	8
GFP-B4G	19.4**	0.001	1
GFP-B5G	22.2**	0.001	3
GFP-B6G	83.9	0.083	7
GFP-B7G	49.6**	0.013	5
GFP-B8G	131.0	0.576	10

Compared with NCG, ^##^ *p* < 0.01. Compared with MCG, *p < 0.05, **p < 0.01.

The effects of GFP on the level of serum CA-125 in endometriosis rats and score for different groups were shown in Table 4. As expected, the level of CA-125 in MCG increased significantly compared with NCG. In addition, the level of CA-125 in the whole prescription group (GFP-A1G) and regularity of recipe composition groups GFP-B1G ∼ GFP-B3G, GFP-B5G, and GFP-B7G ∼ GFP-B8G were apparently decreased, especially for GFP-B2G and GFP-B8G.

**TABLE 4:** CA-125 value change after drug administration.

No.	CA-125(U/ml)	*p*	Score
NCG	0.76	—	—
MCG	1.55^##^	—	—
PG	1.21**	0.001	3
GFP-A1G	1.24*	0.003	4
GFP-B1G	1.30*	0.019	5
GFP-B2G	1.08**	0.000	1
GFP-B3G	1.33*	0.041	8
GFP-B4G	1.41	0.199	9
GFP-B5G	1.32*	0.028	7
GFP-B6G	1.52	0.708	10
GFP-B7G	1.31*	0.017	6
GFP-B8G	1.09	0.000	2

Compared with NCG, ^##^ *p* < 0.01. Compared with MCG, *p < 0.05, **p < 0.01.

According to the synthetic judgment of scores for the volume of ectopic tissue and the level of CA-125 in serum, the effect was best in GFP-A1G, followed by GFP-B2G, GFP-B4G, and GFP-B5G. The results were shown in Table 5.

**TABLE 5:** Total score after administration.

No.	Score		Total score
	Volume	CA-125(U/ml)	
GFP-A1G	2	4	6
GFP-B1G	6	5	11
GFP-B2G	9	1	10
GFP-B3G	8	8	16
GFP-B4G	1	9	10
GFP-B5G	3	7	10
GFP-B6G	7	10	17
GFP-B7G	5	6	11
GFP-B8G	10	2	12

### Gray Correlation Analysis

**FIGURE 4:**
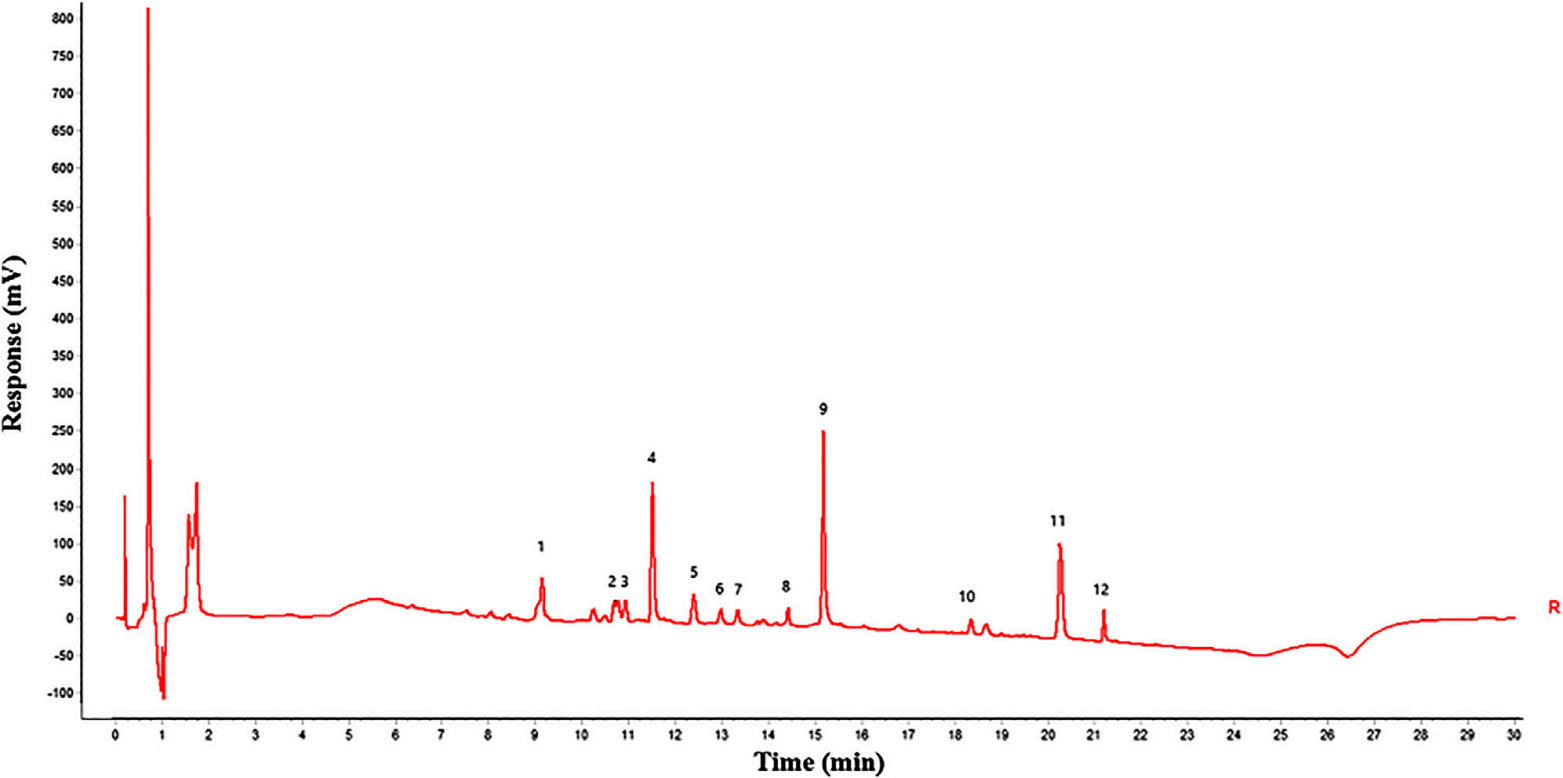
Twelve main peaks in GFP (peaks 1, 4, 9, 10, and 11 were identified as amygdalin, paeoniflorin, pentagalloyl glucose, cinnamic acid, and paeonol, respectively).

Gray correlation analysis (GRA) is a multi-factor statistical analysis method. It is based on the sample data and measured the correlations of each factor via the gray correlation degree. In this work, the spectrum-effect relationship of GFP was studied via the GRA method. A total of 12 main peaks were selected according to the chromatogram of GFP-A1G and GFP-B1G ∼ GFP-B8G (12 main peaks were shown in Figure 4). In order to determine the relationship between the main peaks and the efficacy for the treatment of endometriosis, the total score of each group (Table 5) was taken as the parameter column, and the correlation degree was calculated after the original data was treated with the dimensionless standard. The results of peak area and spectrum-effect correlation analysis were shown in Table 6.

**TABLE 6:** Peak area and correlation analysis results of each chromatographic peak.

No.	Peak area									Correlation degree
	GFP-A1G	GFP-B1G	GFP-B2G	GFP-B3G	GFP-B4G	GFP-B5G	GFP-B6G	GFP-B7G	GFP-B8G	
1	610.59	1,285.68	637.47	559.65	1,286.61	924.14	437.43	2,129.31	520.93	0.782
2	147.32	314.32	0.00	0.00	552.34	0.00	0.00	0.00	134.98	0.632
3	139.02	296.64	113.69	0.00	291.63	0.00	0.00	0.00	0.00	0.600
4	1,007.72	2,311.11	1,178.10	200.93	2,541.38	606.56	0.00	0.00	889.76	0.695
5	263.73	565.60	152.48	268.43	410.46	537.39	0.00	351.43	264.86	0.772
6	123.16	324.05	0.00	0.00	119.81	375.11	0.00	0.00	118.87	0.668
7	163.28	407.49	0.00	105.03	0.00	451.00	0.00	127.30	139.89	0.687
8	149.49	256.58	0.00	0.00	147.44	252.10	0.00	395.07	0.00	0.641
9	1,438.71	3,741.06	409.60	731.72	1,501.84	3,081.52	0.00	120.59	1,181.78	0.694
10	195.96	408.97	288.40	191.91	574.30	408.99	248.49	1,127.25	0.00	0.773
11	1,107.10	1,588.99	0.00	1,035.31	0.00	2,430.15	0.00	0.00	1,165.61	0.672
12	114.40	257.13	0.00	0.00	105.91	205.01	0.00	112.37	130.46	0.736

As shown in Table 6, the correlation degree between 12 main peaks and the total score were greater than 0.6. This result indicated that various constituents in the GFP may be acting in synergy. The ranking of correlation degree was the number of peak 1 > 10 > 5 > 12 > 4 > 9 > 7 > 11 > 6 > 8 > 2 > 3. According to the analysis results of UPLC/Q-Tof-MS/MS, peaks 1, 4, 9, 10, and 11 were identified as amygdalin, paeoniflorin, pentagalloyl glucose, cinnamic acid, and paeonol, respectively. These components could be candidates for the Q-Markers of GFP for endometriosis treatment.

### Molecular Docking Analysis

In our study, a total of 13 candidate compounds present in GFP were molecularly docked with these 41 proteins associated with endometriosis. A total of 9 compounds exerted potential docking with these 41 proteins in Table 7. However, their capabilities of docking were different. Paeoniflorin, amygdalin, paeonol, benzoic acid, gallic acid, pentagalloyl glucose, benzoylpaeoniflorin, and cinnamic acid showed better binding capability than other candidate compounds (all of their docking scores were lower than −5). The results indicated that these 9 components might play a critical role in the anti-endometriosis effect of GFP. Each protein and corresponding compounds with the highest docking scores were shown in Table 7.

**TABLE 7:** Results of docking.

PDB IDs	Compound	-CDOCKER ENERGY	PDB IDs	Compound	-CDOCKER ENERGY
1A9W	Amygdalin	−6.605	4MR8	Paeonol	−6.423
1A28	Benzoic acid	−7.506	4P6X	Paeonol	−6.958
1D5R	Benzoic acid	−5.671	4PIV	Paeoniflorin	−9.25
1FAG	Amygdalin	−7.335	4QP3	Amygdalin	−6.763
1HE8	Gallic acid	−5.766	4WB6	Cinnamic acid	−6.37
1I1Q	Amygdalin	−7.228	5CXV	Paeonol	−5.793
1LQF	Paeonol	−5.284	5F1A	Benzoylpaeoniflorin	−7.735
10JD	Paeonol	−7.356	5I56	Amygdalin	−7.82
1PY2	Paeoniflorin	−5.899	5IC4	Gallic acid	−5.082
1YTZ	Paeoniflorin	−5.654	5IVB	Gallic acid	−5.553
2LSQ	Amygdalin	−5.293	5L36	Paeoniflorin	−6.323
2P2I	Paeonol	−8.103	5MU8	Benzoic acid	−5.135
2P9K	Paeoniflorin	−6.711	5MZA	Paeonol	−4.433
3F5P	Paeoniflorin	−6.785	5UC4	Amygdalin	−7.227
3O96	Paeoniflorin	−10.055	5W5X	Amygdalin	−4.802
3QXY	Pentagalloyl glucose	−8.283	6ATQ	Paeoniflorin	−5.594
3TGX	Paeoniflorin	−6.962	6CM4	Paeoniflorin	−7.35
3UEO	Paeoniflorin	−6.434	6CMJ	Amygdalin	−9.424
4GLP	Benzoic acid	−6.901	6EJE	Amygdalin	−7.592
4IEH	Benzoylpaeoniflorin	−7.22	6MXT	Gallic acid	−8.301
4MQT	Paeonol	−8.00			

## Conclusion

Traditional Chinese herb formulations (TCHF) were a bright pearl in TCM. More and more TCHF were employed in the treatment of chronic disease and difficult miscellaneous disease. Nevertheless, the problem of quality control has been restricting the standardization and modernization of TCHF. Luckily, the proposed Q-Markers improve and guide the quality control of TCM. In spite of the great efforts that had been made, the discovery and screening out of the Q-Marker were still a huge challenge for TCHF.

Based on the above, a gray correlation analysis based Q-Marker discovery strategy was proposed in this study. Compared with other methods to discover Q-Markers, the gray correlation analysis established good relations between components and efficacy and took full advantage of existing data. As a result, five components including amygdalin, paeoniflorin, pentagalloyl glucose, cinnamic acid, and paeonol were screened out as the Q-Markers for the treatment of endometriosis in GFP. Docking experiment with lower binding energies confirmed their binding capability with target proteins. It is promising that the gray correlation analysis could be contributed to Q-marker discovery. Furthermore, this is the first time for gray correlation analysis strategy applied to the discovery of Q-Marker. However, the contribution of each Q-Marker has not been clarified in this study, which needs to be studied for further investigation.

## Data Availability Statement

The raw data supporting the conclusions of this article will be made available by the authors, without undue reservation, to any qualified researcher.

## Ethics Statement

The animal study was reviewed and approved by Huafukang Biotechnology Co., Ltd.

## Author Contributions

JC: conducted the experiment and performance data analysis and wrote the paper. XG, XX, YL, and TR: participated in the experimental process. SL: identified the herbs. CT: conceived, designed, and supported this study and critically revised the paper with CL. All of the authors participated in the study and approved the submitted version of the paper.

## Funding

In particular, this study was supported by the National Science and Technology Major Project of China (2019ZX09201005), Science and Technology Planning Project of Tianjin, China (17YFZCSY00750), and Shandong Science and Technology Project (2016CYJS08A01).

## Conflict of Interest

The authors declare that the research was conducted in the absence of any commercial or financial relationships that could be construed as a potential conflict of interest.
